# ASKING THE PEOPLE WHO MATTER THE MOST: DESIGNING A VALUE-BASED DEMENTIA SPECIALTY CLINIC

**DOI:** 10.1093/geroni/igz038.434

**Published:** 2019-11-08

**Authors:** Alyssa Aguirre, Christopher Ulack, Joel Suarez, Kathleen Carberry, Justin Rousseau, Scott Wallace, Robin Hilsabeck

**Affiliations:** 1 University of Texas Dell Medical School, Austin, Texas, United States; 2 University of Texas, Austin, Texas, United States

## Abstract

This presentation will highlight our research which uses a qualitative methodology to incorporate the voices and experiences of people impacted by dementia into the value-based health model. This model is characterized by a team-based approach as well as the measurement of outcomes. The aim of value-based care is to provide individuals meaningful and compassionate care that helps them achieve the health outcomes that matter most to them. Foundational to creating this person-centered model is the incorporation of the perspectives of individuals with dementia and their care partners. Experience Groups offer an opportunity for those affected by dementia to share their expertise and describe their daily challenges and successes so we are able to learn from their experiences and better understand unmet and unarticulated needs. The findings of this research—consisting of 41 patients and 11 care partners—enabled the development of outcome measurement tools implemented at the clinical level, and the design of a care delivery model that addresses unmet needs. Some of the key findings from the research that have been implemented at the Cognitive Disorders Clinic and that will be highlighted in this poster are: 1. Care partners would like more emotional support from their medical team; 2. Individuals want more information about the trajectory of the disease and an actionable “roadmap-of-care”; 3. Care partners and those with early stage memory loss desire counseling and team-based care versus strictly physician-provided care.

